# Improved Toughness in Lignin/Natural Fiber Composites Plasticized with Epoxidized and Maleinized Linseed Oils

**DOI:** 10.3390/ma13030600

**Published:** 2020-01-28

**Authors:** Franco Dominici, María Dolores Samper, Alfredo Carbonell-Verdu, Francesca Luzi, Juan López-Martínez, Luigi Torre, Debora Puglia

**Affiliations:** 1 Civil and Environmental Engineering Department, University of Perugia, UdR INSTM, Strada di Pentima 4, Terni 05100, Italy; francesca.luzi@unipg.it (F.L.); luigi.torre@unipg.it (L.T.); debora.puglia@unipg.it (D.P.); 2 Instituto de Tecnología de Materiales, Universitat Politècnica de València, Plaza Ferrandiz y Carbonell, 03801 Alcoy-Alicante, Spain; masammad@upvnet.upv.es (M.D.S.); alcarve1@epsa.upv.es (A.C.-V.); jlopezm@mcm.upv.es (J.L.-M.)

**Keywords:** Arboform, epoxidized oil, maleinized linseed oil, toughness, thermal stability

## Abstract

The use of maleinized (MLO) and epoxidized (ELO) linseed oils as potential biobased plasticizers for lignin/natural fiber composites formulations with improved toughness was evaluated. Arboform^®^, a lignin/natural fiber commercial composite, was used as a reference matrix for the formulations. The plasticizer content varied in the range 0–15 wt % and mechanical, thermal and morphological characterizations were used to assess the potential of these environmentally friendly modifiers. Results from impact tests show a general increase in the impact-absorbed energy for all the samples modified with bio-oils. The addition of 2.5 wt % of ELO to Arboform (5.4 kJ/m^2^) was able to double the quantity of absorbed energy (11.1 kJ/m^2^) and this value slightly decreased for samples containing 5 and 10 wt %. A similar result was obtained with the addition of MLO at 5 wt %, with an improvement of 118%. The results of tensile and flexural tests also show that ELO and MLO addition increased the tensile strength as the percentage of both oils increased, even if higher values were obtained with lower percentages of maleinized oil due to the possible presence of ester bonds formed between multiple maleic groups present in MLO and the hydroxyl groups of the matrix. Thermal characterization confirmed that the mobility of polymer chains was easier in the presence of ELO molecules. On the other hand, MLO presence delayed the crystallization event, predominantly acting as an anti-nucleating agent, interrupting the folding or packing process. Both chemically modified vegetable oils also efficiently improved the thermal stability of the neat matrix.

## 1. Introduction

At the present time, the use of eco-sustainable materials is becoming a required condition of worldwide interest. It is known that polymeric materials are derived from fossil fuels and these limited resources can be preserved if, from a sustainable perspective, biobased materials containing the maximum amount of renewable biomass derivatives will be considered. The main concern is related to the possible substitution of materials and products traditionally made from petroleum resources with biobased plastics and composites.

To antedate this need, the German Fraunhofer Institute for Chemical Technology together with Tecnaro GmbH Company studied and developed a new material based on wood components that can be processed as a thermoplastic polymeric material. Arboform^®^ is composed of natural fibers, lignin and additives and can be obtained by using different types of lignin, typically at 30% wt, natural fibers (60 wt %, flax, hemp, sisal, wood) and 10% natural additives (softeners, pigments, processing agents, etc.) that make it a fully biodegradable bioplastic composite known as “liquid wood [1]”. The material properties—biodegradability and recyclability up to ten times without modifications of its features—recommended it to be the near future alternative to various traditional plastic materials [2]. Depending on the quantity of the mixed components, Arboform can be commercially found in three different options: LV3 Nature, F45 Nature and LV5 Nature. These composite materials can be extruded and injected by using the same technologies applied to engineer polymers based on lignin as a matrix and reinforced with natural fibers [3,4,5].

The appearance of Arboform samples is typical of a woody-like material, while the mechanical behavior properties are in the range of the engineering thermoplastics, such as polyamides. However, even if it is characterized by high values for strength and elastic modulus, this composite material shows a drawback in a modest toughness, which restricts its applications as an impact-resistant material [6,7]. The plasticization of a lignin biopolymer has been studied by Bouajila et al. [8], who clearly indicated that mechanisms of lignin plasticization are totally altered in dry and in wet conditions. In detail, they demonstrated that unsaturated lignin (dry lignin, plasticized by low amounts of plasticizers) is better plasticized by molecules that can be involved in H bonds, while hydrated lignin is better plasticized by aromatic molecules with a structure similar to that of monolignols (e.g., vanillin) and by molecules with a solubility parameter similar to that of the matrix [9]. The plasticization of lignin with organic plasticizers was originally reported in 1975 by Sakata and Senju [10], who studied the thermal softening temperatures of lignin plasticized by a variety of plasticizers, including esters of phthalic acid, phosphoric acid and aliphatic acids. Previous examples of combined use of vegetable oils and lignin can be found in Antonsson et al. [11], where low-molecular-weight lignin was used together with a vegetable oil to produce a new hydrophobic lignin derivative similar to suberin, demonstrating the potential use in paper-coating applications (due to its capability to interact well with wood fibers and make paper hydrophobic). However, to the best of the authors’ knowledge, there are no examples of natural additives considered as toughening agents for lignin biopolymer. In the present study, linseed oil was selected as a natural additive able to improve the properties of the composite without compromising its biopolymeric nature. In order to improve compatibility with the matrix, an epoxidized linseed oil (ELO) and a linseed oil with maleic anhydride modification (MLO) were chosen.

## 2. Materials and Methods

A commercial composite material named Arboform^®^ grade L (Tecnaro GmbH, Ilsfeld, Germany), V3 Nature was supplied by Tecnaro GmbH (Ilsfeld, Germany). This thermoplastic material, obtained by biorenewable resources, is characterized by a density of 1.29 g/cm^3^ and a melt volume rate (MVR) (at 190 °C/2.16 Kg, 15 cm^3^/10 min). Epoxidized linseed oil (ELO), supplied by Traquisa S.A. (Barcelona, Spain) with a molecular weight 1.037–1.039 and an epoxy equivalent weight (EEW) of 178 g equiv^−1^, and maleinized linseed oil (MLO) supplied as Veomer Lin by Vandeputte (Mouscron, Belgium) with a viscosity of 10 dPa s at 20 °C and an acid value of 105–130 mg KOH g^−1^ were used as plasticizers.

### 2.1. Processing of Plasticized Compounds

Formulations based on Arboform L, V3 added with ELO and MLO are reported in Table 1. Due to the saturation limit of the matrix with epoxidized oil (miscibility problems between the matrix and this quantity of epoxidized oil during the blending process), it was not possible to produce the formulation with 15 wt % of ELO. Four formulations of Arboform L, 3V plasticized with a content of ELO between 1 wt % and 10 wt % and five using MLO at a percentage between 1 wt % and 15 wt % were produced to compare their properties with the neat matrix.

After drying at 50 °C for 24 h, Arboform^®^ L, V3 was mixed with the modified oils in a co-rotating twin-screw extruder (D = 30 mm; L/D = 20:1) by DUPRA (Alicante, Spain) at a rotation speed of 25 rpm with a temperature profile of 165–170–173–175 °C. After cooling to room temperature, the materials were pelletized and dried at 50 °C for 24 h. All 10 formulations were processed in a Meteor 270/75 injection molding machine (Mateu & Solé, Barcelona, Spain) with a temperature profile of 160–165–170–175 °C and an injection pressure P_inj_ = 60 MPa. Samples for tensile, flexural, impact, heat deflection and dynamic mechanical thermal analysis (DTMA) tests have been manufactured. Standard samples for tensile tests and rectangular samples sizing 80 × 10 × 4 mm^3^ were obtained for the characterization.

### 2.2. Measurements of Mechanical Properties

The effects of ELO and MLO content on mechanical properties were studied by impact tests. All specimens were conditioned, according to ISO 291 [12], at 23 °C and 50% RH before testing and tested in the same conditions. Impact-absorbed energy was measured with a 6 J Charpy pendulum from Metrotec S.A. (San Sebastián, Spain) on unnotched samples according to ISO 179 standard testing [13]. At least five samples for each material were tested.

Flexural and tensile tests were carried out by a universal test machine Ibertest Elib 30 (Ibertest S.A.E., Madrid, Spain) at room temperature. A minimum of five different samples was tested using a 5 kN load cell. The crosshead speed for the tensile tests was set at 10 mm min^−1^ as recommended by ISO 527 standard [14]. An axial extensometer by Ibertest was used to give accurate values of Young’s modulus for each material. Flexural characterization was performed setting the crosshead speed to 5 mm min^−1^ for three points bending test, as suggested by ISO 178 standard [15].

### 2.3. Thermo-Mechanical Characterization

Thermo-mechanical behavior of the mixtures was studied by heat deflection temperature (HDT) tests and by dynamic mechanical thermal analysis (DMTA). The HDT was determined by the A method according to ISO 75 standard [16] that recommends a load of 1.8 MPa and a heating rate of 120 °C min h^−1^. Tests were carried out in a VICAT/HDT station DEFLEX 687-A2 by Metrotec S.A. (San Sebastian, Spain).

DMTA in torsion mode of Arboform L, V3 and its formulations, plasticized with ELO and MLO, was carried out with an oscillatory rheometer AR G2 by TA Instruments (New Castle, DE, USA), equipped with accessory clamps for solid samples. Rectangular samples sizing 40 × 10 × 4 mm^3^ were subjected to a temperature ramp from 30 °C to 100 °C, setting the heating rate at 2 °C min^−1^. The maximum deformation (γ) was at 0.1% and all samples were tested at a constant frequency of 1 Hz. The curves of the storage moduli (G’), the loss moduli (G’’) and their ratio G”/G’ as Tan Delta (tan(δ)) versus the temperature were determined.

Differential scanning calorimetry (DSC) tests were carried out with a TA Instruments Mod. Q200 (TA Instrument, New Castle, DE, USA) calorimeter to determine the effect of ELO and MLO content on the thermal properties of the blends based on Arboform L, V3. For DSC analysis, approximately 10 mg of each sample was placed in a hermetically sealed sample pan after the calibration of the instrument with indium standard. Tests were performed in a three-step cycle: heating, cooling and heating scans, from 25 °C to 180 °C at 10 °C min^–1^. Glass transition temperature (T_g_), crystallization and melting phenomena of the blends were determined.

Thermal degradation behavior of the formulations was evaluated by thermogravimetric analysis (TGA, Seiko Exstar 6300, Tokyo, Japan); around 5 mg samples were used to perform dynamic tests in a nitrogen atmosphere (200 mL min^–1^) from 30 to 600 °C at 10 °C min^–1^. Thermal degradation curves for the Arboform L, V3 and formulations with 5 wt % and 10 wt % modified with each oil were evaluated.

### 2.4. Morphological Characterization

The morphology and the microstructure of the blend samples were observed. Fractured surfaces from impact tests were captured by field emission scanning electron microscopy (FESEM) in a Zeiss Ultra microscope 55 (Oxford Instruments, Oxfordshire, UK) with an accelerating voltage of 2 kV. Fractured surfaces were previously coated with a thin platinum layer in a sputter coater EM MED020 (Leica Microsystems, Wetzlar, Germania).

## 3. Results and Discussion

The study of the effect of the addition of linseed oils modified with epoxidation and maleinization treatment to the commercial biopolymer Arboform grade L, V3 Nature was carried out by a wide characterization of the formulated materials. Since modest ability to absorb shocks is a critical property of Arboform L, V3, it was tried to increase its toughness by adding plasticizers.

**Figure 1:**
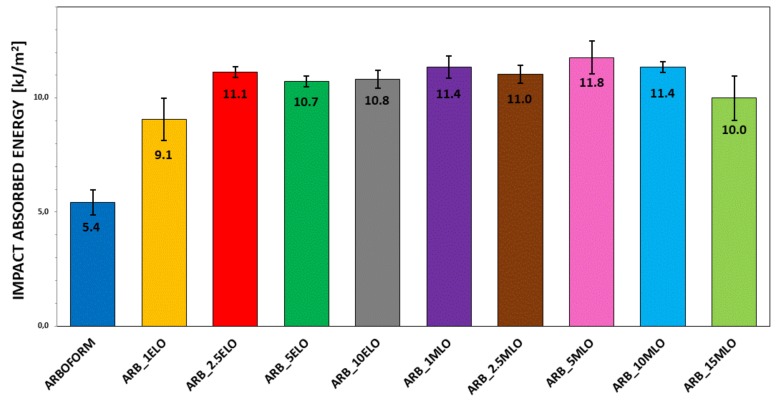
Impact-adsorbed energy for epoxidized (ELO)- and maleinized (MLO)-modified samples based on Arboform L, V3 matrix.

The results of impact tests represent the combination of two effects: on the one hand, fracture resistance associated with the mechanical strength; on the other hand, deformation capability that is directly related to the ductile mechanical behavior. The impact-absorbed energy depends on several factors, such as crack sizes and growth speed, presence of stress concentrators, phase separation, etc. All these factors can modify the overall deformation capacity and, subsequently, the total energy absorbed during deformation and fracture. Impact test results in Figure 1 show a general increase in the impact-absorbed energy for all the samples modified with bio-oils. Absorbed energy values of neat Arboform L, V3 (5.4 kJ/m^2^) increased to 11.1 kJ/m^2^ with an improvement of 105% with ARB_2.5MLO. Formulations ARB_5MLO and ARB_10MLO show values of impact-adsorbed energy of 10.7 kJ/m^2^ and 10.8 kJ/m^2^, respectively, corresponding to a percentage increase of 98% and 100% with the addition of 5 and 10 wt % of MLO. The addition of 2.5% wt. of ELO to Arboform is enough to double the quantity of absorbed energy and this value slightly decreased for samples containing 5 and 10 wt % of ELO. A similar result is also obtained with the addition of MLO, but in this case, 1 wt % was enough to appreciate an improvement of the toughness of the materials. In the case of MLO-modified samples, the absorbed energy values result doubled respect to the matrix for all the formulations. The best result of impact resistance is appreciated in the material with 5 wt % of MLO, with an improvement of 118%, after which a slight decrease is observed. Similar behavior was also reported by others [2,17,18].

It was found that the addition of synthetic compatibilizers, such as polymeric methylene diphenyl diisocyanate (PMDI) at low concentrations (1 wt %), produces an increase of the absorbed energy up to 92% and then progressively decreases, increasing the PMDI content to 2 wt %. Similarly, to what was established for PMDI, high quantities of modified oils can produce a phase separation that does not contribute to the improvement of impact resistance [19,20].

**Figure 2:**
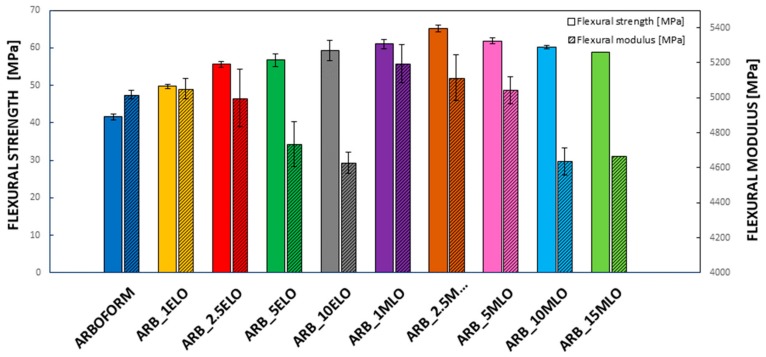
Comparative bar plot of flexural strength and flexural modulus for neat Arboform L, V3 and its formulations with ELO and MLO.

The results of the flexural tests (Figure 2) show that the addition of ELO produces a progressive increase in tensile strength as the percentage of oil increases; simultaneously, a reduction in the flexural modulus is observed when the epoxidized oil content increases. The effectiveness of ELO as a coupling agent, as well as a plasticizer, with biopolymers and lignin-based fillers, was already demonstrated [21,22,23]. The progressive increase in strength with the ELO content is attributable to the good compatibility between lignin and fibrous reinforcement contained in the Arboform. Above 2.5 wt % of ELO, the plasticizing effect of the epoxidized linseed oil molecules enables polymer chain mobility so that the modulus decreases [24].

The addition of maleinized oil produces a significant improvement in flexural strength, which exceeds the strength of the matrix for all formulations added with MLO. The ARB_2.5MLO formulation shows a flexural strength of 65.2 MPa compared to 41.6 MPa of the Arboform, with an increase of 56.7% [19,25,26].

Formulations with an MLO content of 5 wt % and above show a slight decrease in the flexural strength, essentially due to an anti-plasticizing effect. Some authors have described this anti-plasticizing effect with a potential saturation of the plasticizer [20]. Gutierrez-Villareal et al. experimentally found this phenomenon when citrate esters were used to plasticize poly (methyl methacrylate) (PMMA) [27] with a low concentration of plasticizer. Vidotti et al. have guessed that the anti-plasticizing effect may be due to a reduction in the free volume [28] so that when the free volume is filled by the plasticizer, the phenomenon may appear. The increase in strength, associated with the anti-plasticization phenomenon, can also be explicated by considering the trend in crystallinity as the plasticizer enhances chain mobility, thus the crystallization tendency is favored. The anti-plasticizing effect depends on the molecular weight, the concentration of the plasticizer and the characteristics of the polymer matrix, and it is specific for each polymer–plasticizer system [29]. Elastic modulus increased for the formulations added with 1, 2.5 and 5 wt % of MLO. The drop of the flexural modulus for the formulations with higher percentages of modified oils is essentially related to an excess of oil molecules, which does not bind directly to the polymeric matrix. Plasticizer excess can have a negative effect on homogeneity by forming a dispersed phase in the main matrix. A high plasticizer content produces intense plasticizer–plasticizer interactions, leading to a phase separation [30]. Chieng et al. concluded that, due to the high plasticizer content, only a small part is directly in contact between the interface area, while the excess is dispersed in the polymer matrix [31,32].

The results of the tensile tests (Table 2) show a slight decrease in the values of the elastic modulus, which progresses with the increase in the content of both modified oils. A general increase in tensile strength is obtained for all the formulations produced: this increase is significant for the formulations with the higher epoxidized oil contents (5, 10 wt % ELO), while the higher values of the tensile strength are obtained with lower percentages of maleinized oil (1, 2.5, 5 wt % MLO).

The effect of high ELO contents compared to low quantities of MLO is justified by the different operating principles. Lignin is considered a potential substitute for bisphenol A in the synthesis of epoxy resins due to the presence of hydroxyl groups (in particular of phenolic hydroxyl group) in the lignin structure. However, structure and steric hindrance limit epoxidation reactions. The reduced reactivity with the epoxy groups requires high quantities of epoxy oil for a binding effect to be detected [33]. Furthermore, for high quantities of ELO, a self-polymerization phenomenon of linseed oil cannot be excluded [34]. Essentially, the best results are obtained for formulations with higher ELO content. It was observed that ester bonds can be potentially formed between multiple maleic groups present in MLO and the hydroxyl groups of green composites, as in the case of Arboform. This reaction could induce an effective stress transfer between lignin and fibrous components of the composite material, improving their mechanical properties [25].

Moving the composition from 5 to 10 wt % of MLO, a reduction in both strength and modulus was observed. This negative effect can be related to the phase separation caused by an excessive concentration of MLO in the composite. Excess MLO molecules, which do not form direct bonds with the composite molecules, are placed between the biopolymer chains and act as a lubricant with a greater influence on the mobility of the chain. This result, together with previous mechanical tests, suggests that optimal and mechanically balanced performance is obtained for MLO contents of about 5 wt % The best results of tensile strength are obtained with the formulations containing 5 wt % of each oil. The values of the strains at the break of the materials modified with epoxidized oil show modest improvement, while a slight increase in strain values at the break was found for materials modified with MLO. The formulations with the content of each oil between 5 wt % and 10 wt % show the best performance in terms of tensile elongation at break.

**Figure 3:**
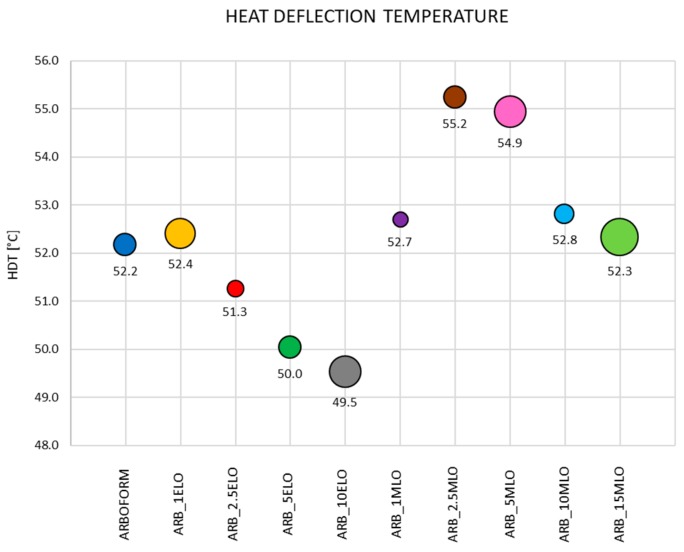
Bubble chart of the Heat Deflection Temperature test.

*HDT measurements:* The bubble chart in Figure 3 represents the deflection temperature distributions of the materials added with the modified linseed oils. The center of the bubble indicates the average value of the HDT, and the diameter size is its standard deviation. The initial HDT value for the Arboform composite is 52.2 °C, which shows that moderate temperatures lead to material softening. The addition of 1% by weight of ELO produces a slight increase at 52.4 °C in HDT, which is negligible when considering the standard deviation, compared to the Arboform L, V3 matrix. Higher percentages of epoxidized oil cause a gradual decrease in characteristic deflection temperatures. In relation to the evolution of HDT in terms of ELO wt %, the plasticization provided by ELO leads to softer and more flexible materials, therefore the values of the deflection temperatures decrease with increasing ELO content up to values of 51.3 °C, 50.0 °C and 49.5 °C for an ELO content of 2.5, 5 and 10% by weight, respectively. Epoxidized linseed oil acts as a plasticizer, producing a visible softening effect, which is more evident by the application of external loads low-temperature values. Mobility of polymer chains is easier in the presence of ELO molecules, since these reduce the intermolecular attraction forces between polymeric macromolecules [35]. The stability of HDT for the ARB_1ELO formulation can be explained, in addition to the low oil content, by a slight increase of crystallinity with ELO content, as the plasticizer allows more intense polymer chain motion. The presence of ELO plasticizer allows chain mobility, and this has a positive effect on crystallinity because polymer chains can rearrange to better ordered/packed structures. For ELO contents greater than 1%, the plasticizing effect prevails on crystallization and the benefit on HDT becomes irrelevant [24].

The formulation containing 1 wt % of MLO shows only a slight increase in HDT with a narrow dispersion of results. The use of maleinized oil produces an increase in the deflection temperature at the percentages between 2.5 and 5 wt %, to 55.2 °C and 54.9 °C, respectively, while higher percentages cause the drop of HDT to values close to the Arboform matrix deflection temperature. This behavior can be explained by the compatibilizing effect of MLO, which, thanks to the presence of maleic groups, binds easily to the hydroxyl groups of the lignin and the Arboform natural fiber reinforcements, forming bonds that stabilize the composite; this effect, together with the anti-plasticizing effect, is effective for MLO contents up to 5 wt %. For higher quantities of maleinized oil (10–15 wt %), the plasticizing effect becomes predominant by exerting a lubricating action by distancing the polymer chains, as the MLO molecules are positioned between the molecules of the matrix weakening the polymer–polymer interactions (hydrogen bonds, van der Waals or ionic forces), thus increasing the mobility of the chains. This effect produces greater deformability, which results in a decrease of HDT for high MLO contents [25,34,36].

**Figure 4:**
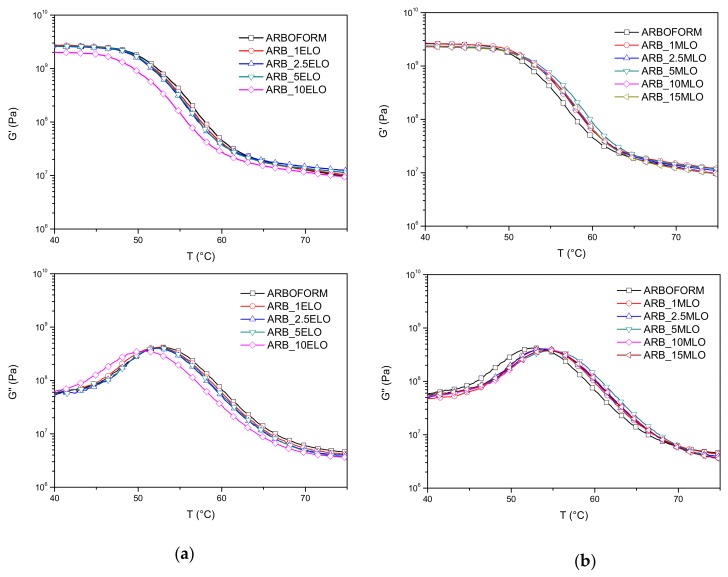
Curves of storage (G′) and loss (G″) moduli of (**a**) ELO-added and (**b**) MLO-added formulations.

*DMTA analysis:*Figure 4a shows the results of DMTA analysis for the Arboform material modified with ELO. Curves of the storage modulus (G’) show a deflection between 50 °C and 60 °C at the glass transition temperature of the reference matrix. In general, the trend for G’ is similar, except for the G’ curve of ARB_10ELO formulation, which shows slightly lower values (in accordance with the results of the moduli obtained from the tensile tests). On the other hand, the higher value for G’ was found for the of the ARB_1ELO formulation, confirming the results of the HDT tests. The crve of ARB_10ELO loss modulus (G”) highlights a shift toward lower temperatures, while the G” curve of ARBOFORM shows the best thermal stability of the set. Peak values of the tan curves (not reported here) show the tendency to progressively decrease in intensity with increasing ELO content [37,38].

In Figure 4b, the results of the DMTA analysis carried out for the materials with increasing content of MLO show that all the formulations have comparable storage moduli in the temperature range between 40 °C and 50 °C; above this range, the modified materials show improved G’ values when compared to the reference matrix. The formulation ARB_5MLO shows the highest modulus value of this material set. All the G” curves of the MLO-added materials show a shift toward higher temperatures. The improvement of the thermal mechanical performance of the materials containing MLO is also confirmed by the increase in T_g_. The T_g_ values, calculated as the maximum peak of the tan (δ) curves (Table 3), show an increase in the glass transition temperatures of all the compounds modified with MLO. The highest value is obtained for the formulation with 5 wt % of MLO, in accordance with HDT tests.

**Figure 5:**
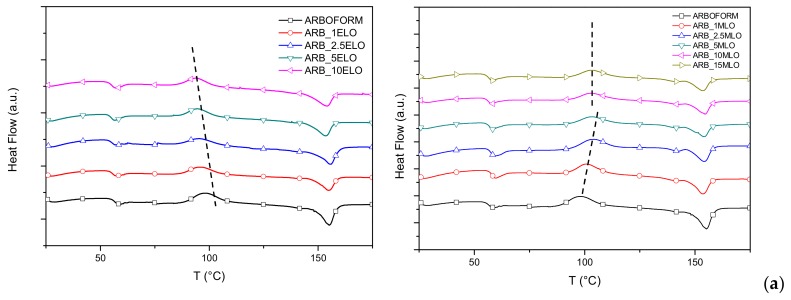

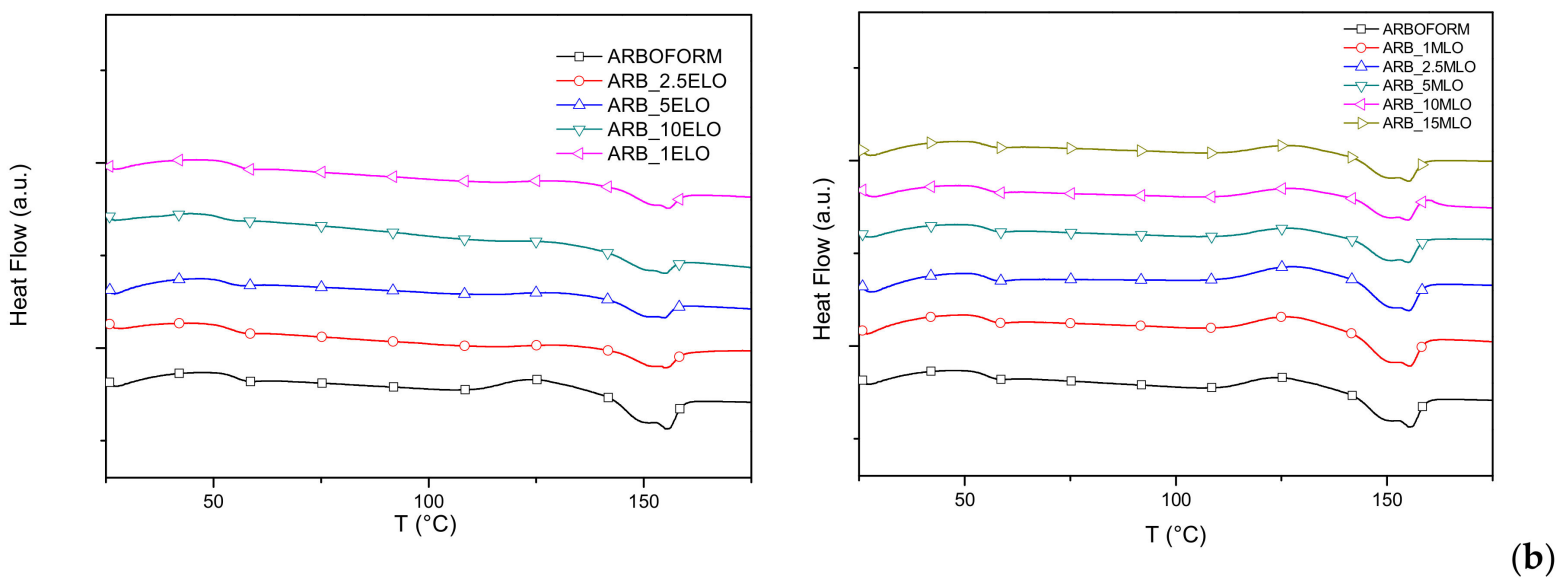
DSC curves (1st heating run (**a**) and 2nd heating run (**b**)) of ELO (left) and MLO (right) added formulations.

*DSC analysis:* Results of thermal characterization for Arboform formulations containing different amounts of ELO- and MLO-modified oils are reported in Figure 5. Other than glass transition events, two main peaks appeared on the DSC thermograms due to an exothermic cold crystallization and an endothermic melting transformation [6,39]. Both of these phenomena can be attributed, in our opinion, to a polylactic acid (PLA) fraction (probably added to improve the workability of liquid wood). The presence of PLA is unequivocally confirmed by the melting peak observed in the neat sample of Arboform, which falls in the melting range of pure PLA [40].

Table 4 shows the results of T_g_, T_cc_ and T_m_, for the studied materials. The literature reports that the MLO has an effect on the reduction of the glass transition temperature for PLA based formulation [41]: in our case, we noticed that T_g_ values remain constant and comparable to the reference material, with no significant differences between MLO and ELO modified formulations. A double melt peak was identified in the formulations (more evident in the second heating scan) due to the formation of non-perfect small crystals that melt at lower temperatures, causing the formation of a small peak [42]. Regarding the cold crystallization temperature (T_cc_), a clear effect was indeed found in every composite containing the modified oils. As seen in Figure 5 and the values in Table 4, the addition of ELO provides a decrease in T_cc_ of Arboform formulations. In particular, ELO favors crystallization due to increased chain mobility, owing to a plasticization effect that allows crystallization to occur with lower energy content. On the other hand, MLO presence delayed the crystallization event: it could be that chain extension, branching or cross-linking of polylactic chains can be promoted, showing a moderate increase in elongation at break in combination to a significant improvement of the mechanical resistant properties (both tensile modulus and strength). This macromolecular change can be based on the fact that the cold crystallization peak was broader (or even disappeared) as the MLO content increased and the melting peak slightly shifted to lower temperatures. In particular, the T_cc_ of Arboform formulations with 1 wt % and 2.5 wt % MLO moved up to 100.7 °C and 103.8 °C, respectively, while the neat matrix showed a T_cc_ of 97.2 °C. A similar T_cc_ was observed for formulations with 5, 10 and 15 wt % MLO with values of 102–103 °C [43]. This suggests a break of the crystalline structure in the polymeric phase, so the shift in the cold crystallization process can be related to the network formation of PLA molecules of higher molecular weight that inhibits chain motion during packing and rearrangement. While one can consider that ELO acts as a plasticizer in the green composites, typically increasing volume and reducing polymer–polymer interactions, MLO particles predominantly acted as an anti-nucleating agent, interrupting the folding or packing process of the predominant PLA chains.

**Figure 6:**
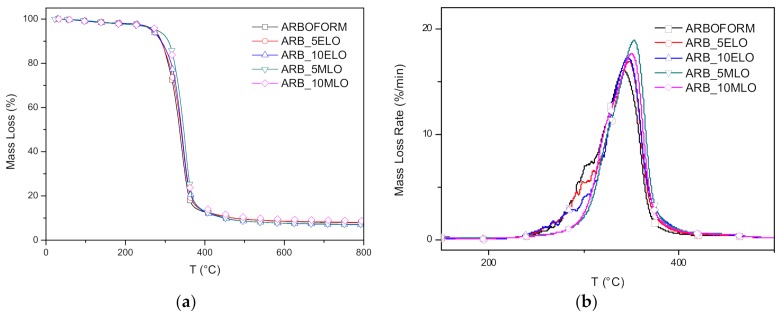
TG (**a**), DTG (**b**), DSC curves of ELO- and MLO-modified Arboform formulations.

*Thermogravimetric analysis:* Results of TGA analysis carried out on materials added with 5 wt % and 10 wt % of the two modified oils (Figure 6) shows that the epoxidized oil confers good thermal stability in the temperature range between 160 °C and 240 °C in comparison with a neat matrix. An increase in thermal stability in materials modified with MLO above 260 °C is to be noted. The derivative curves of thermogravimetric test show an increase in the temperatures of the main peaks of the modified materials with respect to the reference matrix. The highest values for each oil are obtained with 5% by weight: the peak of the matrix at 341 °C moves to 347 °C with the epoxidized oil and it rises to 353 °C with the oil with maleic modification. This result indicates an effective bond of modified oils with the matrix. It is well known that, owing to their high thermal stability, vegetable oils and their chemically modified derivatives can be used as thermal stabilizers in polymers, i.e., poly (vinyl chloride), in addition, it has been also recently reported that EVOs and other vegetable oil-derived materials can efficiently thermally stabilize polylactic based formulations [44]. Linear chain extension, branching and/or, even more intensely, cross-linking effects of MLO are responsible for the achieved thermal stabilization. This is related to the fact that these phenomena are considered to counteract chain scission, potentially leading to a more branched structure.

**Figure 7:**
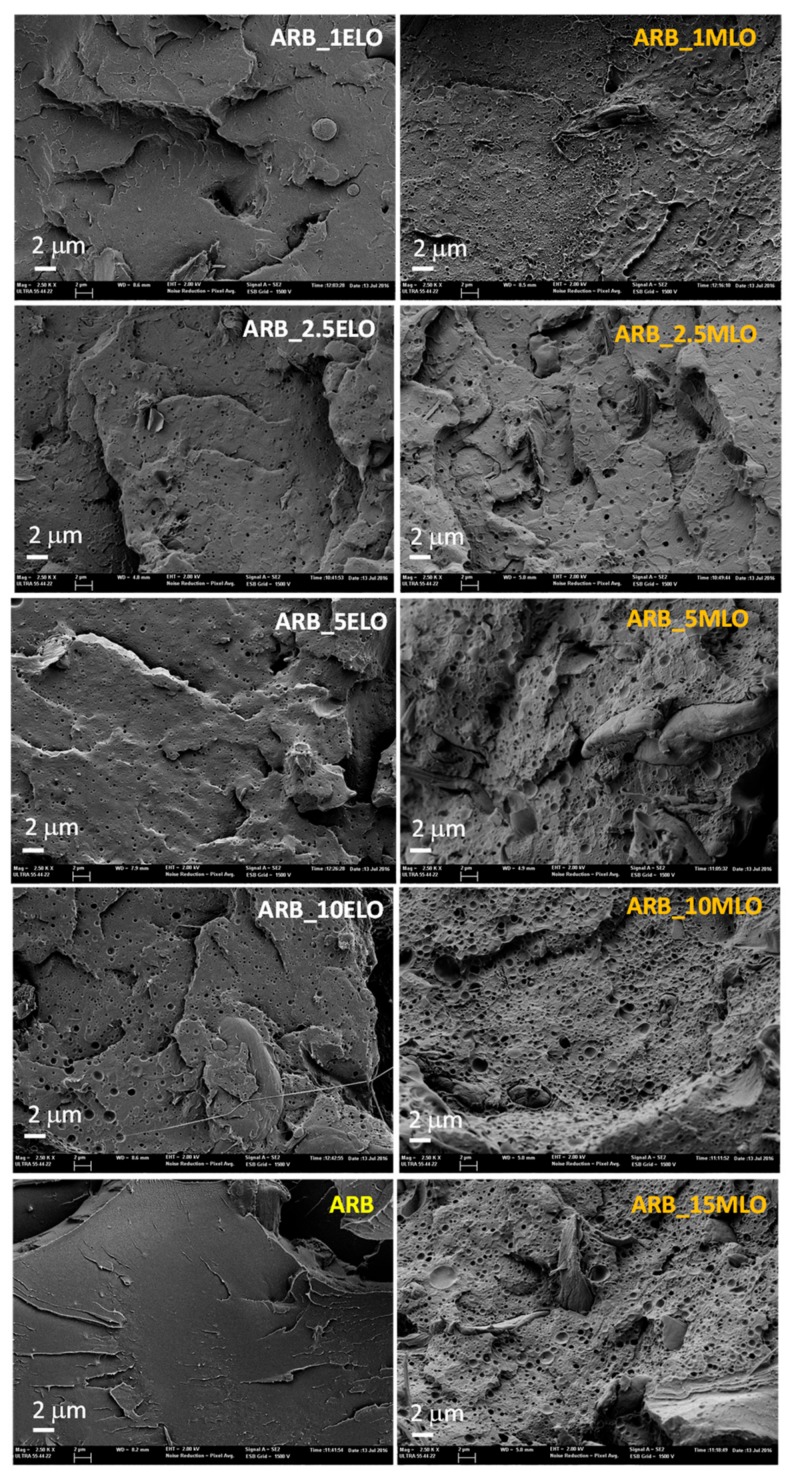
FESEM micrographs of fractured surfaces of ELO- and MLO-modified Arboform formulations.

Morphological analysis: Scanning electron microscopy (SEM) micrographs of fractured surfaces for the Arboform formulations are shown in Figure 7.

It can be observed that phase separation has already occurred at low oil contents, highlighting surfaces with submicrometric sized cavities formed upon solidification of the matrix due to the presence of micro-drops of oil. Arboform surface shows a brittle fracture with some radial cracks typical of materials characterized by low deformation and poor toughness. The neat matrix surface appears smooth and relatively flat surface with low roughness, while a considerable increase in the number of round shape irregularities and/or microvoids on the surface fractures appeared in the case of modified formulations [45]. The addition of increasing amounts of the different vegetable oils (VOs) provides some differences: in particular, the Arboform blend compatibilized with 1 wt % ELO shows a smooth fracture surface similar to that of the neat matrix, while Arboform compatibilized with 1 wt % of MLO shows enhanced presence of cavities/voids.

Phase separation has been reported for vegetable oil-derived additives over 5 wt % as small spherical domains due to excess plasticizer/compatibilizer [46]. The fractured surface of the ELO compatibilized Arboform blend shows strong heterogeneity, which can be related to phase separation, while the MLO compatibilized blends offer a more homogeneous fracture surface, which could be responsible for the maximum achieved elongation at break (see Table 2). Improved miscibility for MLO-modified Arboform formulations can be related to a similar solubility parameter between MLO and the biopolyester contained in the Arboform matrix, thus allowing interactions between them. It has been reported that chemically modified vegetable oils have solubility parameters close to PLA, specifically epoxidized compounds derived from soybean oil achieved a solubility parameter of 16.70 MPa^1/2^ [47], while maleinized oil showed a value of 19 MPa^1/2^, quite similar to the solubility parameter of PLA, which has been reported to be in the range 19.5-22.0 MPa^1/2^ [48,49].

## 4. Conclusions

The addition of environmentally friendly plasticizers derived from vegetable oils, epoxidized (ELO) and maleinized linseed oil (MLO) at relatively low contents (0–15 wt %) led to a significant increase in mechanical properties, such as impact-absorbed energy, tensile and flexural strength, proving that these attractive additives can provide plasticization to brittle polymers and also improve compatibility in immiscible or partially miscible polymer blends. In detail, ARB_5ELO and ARB_5MLO showed an increase, respectively, of +98% and 118% of impact-absorbed energy, +39% and +43% of tensile strength and +37% and +49% of flexural strength. The best results in terms of impact-absorbed energy were obtained with ARB_5MLO (+118%), also tensile (+43%) and flexural strength (+49%), which were resultantly positively affected. In addition, the natural origin of the vegetable oils represents an environmentally effective solution to progress in the preparation and commercialization of industrial formulations based on biopolymers and biopolymeric blends.

## Figures and Tables

**Table 1:** Composition and labeling of the formulations with different modified oils

Reference	Arboform L, V3, wt %	ELO, wt %	MLO, wt %
ARBOFORM	100	0	0
ARB_1ELO	99	1	0
ARB_2.5ELO	97.5	2.5	0
ARB_5ELO	95	5	0
ARB_10ELO	90	10	0
ARB_1MLO	99	0	1
ARB_2.5MLO	97.5	0	2.5
ARB_5MLO	95	0	5
ARB_10MLO	90	0	10
ARB_15MLO	85	0	15

**Table 2:** Result of the tensile test made on ELO- and MLO-modified materials.

Reference	Tensile Modulus [MPa]	Tensile Strength [MPa]	Strain at Break [%]
ARBOFORM	5520 ± 270	23 ± 2	2.1 ± 0.3
ARB_1ELO	5260 ± 400	27 ± 2	2.4 ± 0.2
ARB_2.5ELO	5200 ± 370	28 ± 3	2.6 ± 0.3
ARB_5ELO	4860 ± 410	32 ± 2	2.9 ± 0.2
ARB_10ELO	4730 ± 310	31 ± 2	3.1 ± 0.3
ARB_1MLO	4900 ± 660	32 ± 4	3.0 ± 0.3
ARB_2.5MLO	4760 ± 220	32 ± 2	3.3 ± 0.4
ARB_5MLO	4660 ± 180	33 ± 1	4.2 ± 0.7
ARB_10MLO	4320 ± 310	30 ± 3	3.8 ± 0.5
ARB_15MLO	4500 ± 350	27 ± 1	3.6 ± 0.3

**Table 3:** Values of glass transition temperature, calculated at the maximum of the tan(δ) curve.

Reference	T_g_ (°C)
ARBOFORM	59.1
ARB_1MLO	58.8
ARB_2.5MLO	58.4
ARB_5MLO	58.4
ARB_10MLO	57.5
ARB_1MLO	60.1
ARB_2.5MLO	60.4
ARB_5MLO	61.4
ARB_10MLO	61.0
ARB_15MLO	60.8

**Table 4:** Thermal parameters from DSC analysis of ELO- and MLO-modified Arboform formulations.

	1st Heating						Cooling	2nd Heating					
	T_g_ [°C]	T_cc_ [°C]	ΔH_cc_ [Jg^−1^]	T_m’_ [°C]	T_m’’_ [°C]	ΔH_m_ [Jg^−1^]	T_g_ [°C]	T_g_ [°C]	T_cc_ [°C]	ΔH_cc_ [Jg^−1^]	T_m_ [°C]	T_m’’_ [°C]	ΔH_m_ [Jg^−1^]
ARBOFORM	55.5	97.2	16.0	140.5	154.3	22.7	46.4	55.3	124.9	7.3	148.6	154.9	13.9
ARB_1ELO	55.1	97.3	18.7	141.1	154.7	27.3	47.8	54.7	130.1	3.7	150.4	155.4	9.4
ARB_2.5ELO	54.7	93.7	18.7	140.5	154.2	27.5	46.4	54.0	132.1	3.8	150.7	155.6	6.6
ARB_5ELO	54.4	96.7	18.5	140.2	154.4	28.1	46.4	54.0	130.0	4.0	151.4	154.9	6.8
ARB_10ELO	54.3	95.5	19.0	140.0	154.9	29.0	47.3	52.0	131.1	4.1	151.6	155.2	7.0
ARB_1MLO	57.7	100.7	18.5	140.7	153.6	25.2	47.3	55.5	126.6	12.6	151.4	155.4	14.0
ARB_2.5MLO	57.6	103.8	21.2	142.1	154.0	22.9	47.7	55.6	127.5	12.6	152.2	155.0	13.3
ARB_5MLO	57.2	102.6	21.8	142.8	154.3	24.8	48.4	55.8	127.1	11.6	152.4	155.5	14.1
ARB_10MLO	56.6	102.8	21.6	143.0	154.4	26.9	47.2	55.4	127.0	13.9	150.9	155.0	13.8
ARB_15MLO	56.2	103.2	22.8	142.0	153.4	30.5	46.5	55.3	127.2	15.2	148.6	155.0	14.2
